# WoodenCube: An Innovative Dataset for Object Detection in Concealed Industrial Environments

**DOI:** 10.3390/s24185903

**Published:** 2024-09-11

**Authors:** Chao Wu, Shilong Li, Tao Xie, Xiangdong Wang, Jiali Zhou

**Affiliations:** School of Mathematical Sciences, Zhejiang University of Technology, Hangzhou 310023, China; wuchao@zjut.edu.cn (C.W.); 211122090017@zjut.edu.cn (S.L.); 201806110122@zjut.edu.cn (T.X.); wxd_jy@163.com (X.W.)

**Keywords:** complex industrial scenes, robotic grasping, loss function design, an automatic annotation method, attention mechanisms

## Abstract

With the rapid advancement of intelligent manufacturing technologies, the operating environments of modern robotic arms are becoming increasingly complex. In addition to the diversity of objects, there is often a high degree of similarity between the foreground and the background. Although traditional RGB-based object-detection models have achieved remarkable success in many fields, they still face the challenge of effectively detecting targets with textures similar to the background. To address this issue, we introduce the WoodenCube dataset, which contains over 5000 images of 10 different types of blocks. All images are densely annotated with object-level categories, bounding boxes, and rotation angles. Additionally, a new evaluation metric, Cube-mAP, is proposed to more accurately assess the detection performance of cube-like objects. In addition, we have developed a simple, yet effective, framework for WoodenCube, termed CS-SKNet, which captures strong texture features in the scene by enlarging the network’s receptive field. The experimental results indicate that our CS-SKNet achieves the best performance on the WoodenCube dataset, as evaluated by the Cube-mAP metric. We further evaluate the CS-SKNet on the challenging DOTAv1.0 dataset, with the consistent enhancement demonstrating its strong generalization capability.

## 1. Introduction

With the rapid development of warehousing, logistics, internet, and artificial intelligence technologies, robots have been widely applied in various industrial scenarios [1,2,3,4,5]. As advanced manufacturing demands increasingly higher performance, industrial robotic arms are no longer limited to simple pick-and-place tasks [6,7,8,9]. They now also need to quickly detect object types and accurately localize them to efficiently complete grasping tasks [10,11]. To mitigate the high costs associated with depth cameras, more and more manufacturers have turned to RGB cameras for object recognition and localization [12]. However, a key challenge in this transition is improving the accuracy of object detection using RGB cameras [13]. Some researchers have attempted to directly apply existing object-detection algorithms [14,15], such as convolutional neural networks (CNNs [13]), to the task of object recognition for robotic arms, combined with hand–eye calibration techniques [16] (e.g., eye-in-hand or eye-on-hand) to facilitate grasping; while traditional object-detection algorithms perform well in tasks where targets are clearly visible with high contrast [17,18,19], they face difficulties in scenes where foreground and background textures are similar. In 2020, Fan et al. conducted the first systematic study on concealed object detection (COD) [20], providing a crucial foundation for addressing object detection in complex environments. This pioneering work has subsequently garnered increasing attention from scholars [21,22,23]. However, existing COD tasks predominantly focus on natural images [20,24,25,26], with a scarcity of similar tasks and datasets in the industrial domain. This shortage severely limits the capability of robotic arms to accurately identify and grasp target objects in complex industrial environments, thereby constituting a critical challenge in industrial automation processes.

Since robotic arms require precise knowledge of both the center coordinates and the rotation angle of the target for successful grasping, traditional mAP, a key evaluation metric in object detection, may be insufficient. mAP evaluates detection accuracy by first calculating the IoU (Intersection over Union) [27] between predicted and ground truth bounding boxes, then determining the accuracy based on a given IoU threshold. However, if the center pixels of the predicted and ground truth boxes are roughly the same but the angle differs significantly, mAP may still classify the detection as accurate. For robotic arms, such rotational discrepancies are critical, as they directly affect the precision and stability of the grasp process. Many researchers have proposed improved algorithms based on IoU [28,29,30]. One innovative approach involves modeling the rotated bounding box using a 2D Gaussian distribution [31]. By calculating the Gaussian Wasserstein distance, this method approximates the loss incurred by the non-differentiable rotational IoU, aligning model learning more closely with accuracy measurement and resolving issues related to loss inconsistency. Nevertheless, a notable challenge emerges in the context of square objects, as their Gaussian distribution reduces to a normal distribution with a covariance of 0, resulting in a constant loss value of 0, regardless of changes in IoU.

**Figure 1:**
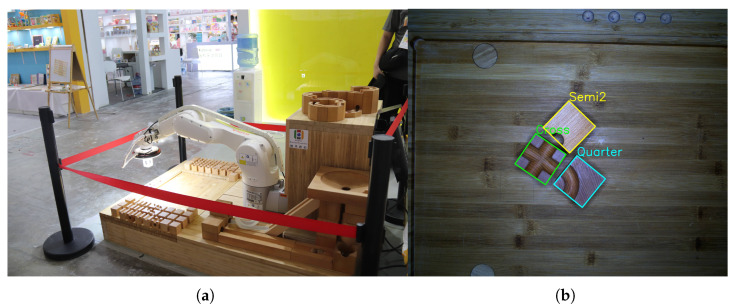
(**a**) A faceted rail track wooden cube scene, where the floor and the blocks share the same material, and the blocks are randomly arranged on this wooden board. (**b**) A bird’s-eye view shows examples of three different types of blocks, with the blocks to be detected having a texture very similar to that of the baseboard.

To solve these issues, we introduce WoodenCube, a dataset specifically focused on concealed object detection in industrial robotic arm grasping scenarios. This dataset was collected in the facade track wooden cube scene, as shown in Figure 1a, featuring up to 10 types of wooden blocks randomly scattered on the surface with similar textures. Figure 1b illustrates three different types of wooden blocks. To enhance annotation efficiency and accuracy, we propose a semi-automatic annotation method based on SAM [32], called Cube Semi-automatic Segment Anything (CS-SAM). Given the high texture similarity between the foreground and background in industrial settings, we initially provide a horizontal prediction box as the initial detection range for SAM. We then use the SAM model to obtain a high-precision mask. Further, by combining actual robotic arm operations and hand–eye calibration techniques, we can directly obtain an initial detection range. Integrating this technique with the SAM model significantly improves the efficiency and accuracy of the annotation process.

Inspired by the work of Yang et al. [31] and Jeffri M. Llerena et al. [33], we introduce a novel evaluation method—G/2-ProbIoU, tailored for scenarios with a large number of square-like features in practical applications. We firstly convert the rotated bounding box into a 2D Gaussian distribution, and then we perform a carefully designed stretching operation on the Gaussian distribution to ensure it does not become isotropic. Finally, by calculating the Bhattacharyya coefficient between the two Gaussian distributions, we accurately measure the overlap between the two bounding boxes. Based on the G/2-ProbIoU threshold, we propose a metric called Cube-mAP to comprehensively evaluate the detection and recognition performance of rotated bounding boxes. By applying this novel Cube-mAP evaluation metric to current models, we can more precisely measure model performance in object detection tasks and efficiently guide model performance optimization. Especially in handling rotated objects, Cube-mAP can capture rotational errors that traditional evaluation methods might overlook, leading to significant performance improvements in this crucial dimension. This improvement enhances the model’s practicality while establishing a solid foundation for future research and applications in the field.

In exploring robotic arm grasping tasks, we inevitably encounter challenges in accurately estimating the poses of target objects. Although existing object-detection models perform well within certain frameworks [34,35,36], they often struggle with predicting rotational components, which is a key factor restricting precise grasping. Inspired by concepts from LSKNet [37] and feature pyramid networks, we propose a multi-scale pyramid-shaped large-kernel convolution network, the Cross-Scale Selective Kernel Network (CS-SKNet). By expanding the network’s receptive field, CS-SKNet captures the rich and distinct texture features that are present in the scene, thereby leveraging the multi-scale feature extraction advantages of FPN and additionally enhancing the model’s performance in complex scenarios.

The core function of the CS selection sub-block in the network is to dynamically adjust its receptive field according to actual needs. This functionality is primarily achieved through the uniquely designed CS-SK core module, which integrates pyramid-shaped large-kernel convolutions and a spatial kernel selection mechanism, allowing the network to flexibly adjust its spatial resolution and contextual information capture capability when confronted with inputs of varying scales and complexities. The MLP sub-block, on the other hand, plays a crucial role in channel mixing and feature refinement within the neural network. It comprises an initial convolution layer, followed by group convolution layers, the GELU activation function, and an additional convolution layer. Collectively, these operations not only enhance the interaction and fusion between features but also refine feature representations through nonlinear transformations, significantly improving the quality and diversity of the resulting feature maps.

The main contributions of our paper are summarized as follows:

- This paper addresses the challenges of industrial robots in recognizing and localizing objects in complex scenes: We introduce the WoodenCube dataset, which explores the detection of objects with textures similar to the background in industrial scenarios. The dataset comprises 5113 densely annotated images of 10 different types of blocks, significantly improving annotation efficiency and accuracy through a semi-automatic annotation method, CS-SAM.
- To tackle the issue that mAP cannot effectively evaluate rotation scales due to the covariance of Gaussian distribution for nearly square features being zero, we propose G/2-ProbIoU and define Cube-mAP based on this function to more accurately assess the detection performance of models on cube-like objects.
- To address the challenges of traditional convolutional methods in distinguishing objects with similar textures, we design a multi-scale pyramid-shaped large-kernel convolutional network, CS-SKNet. This network expands the receptive field to capture strong texture features in the scene, retaining the multi-scale feature extraction advantages of FPN while further enhancing the model’s performance in complex scenarios.
- Extensive experiments conducted on the WoodenCube dataset and DOTAv1.0 dataset demonstrate the competitive performance of the proposed CS-SKNet model in terms of rotated object detection accuracy. On the WoodenCube dataset, our model achieved a Cube-mAP score of 72.64%. On the DOTAv1.0 dataset, our model achieved an mAP of 79.17% while maintaining low parameter count and FLOPs.

## 2. Related Works

### 2.1. Grasp Detection

Object grasping is a fundamental problem with widespread applications in industry, agriculture, and services. Although traditional manual teaching methods can achieve efficient task execution, they become impractical when frequent changes to robot programming are required due to environmental or other factors [38]. Among deep-learning-based grasp-detection methods, the use of RGB-D image input to detect graspable rectangles has gained popularity. Lenz et al. [39] proposed a cascaded network method that first eliminates unlikely grasps, then uses a larger network to evaluate the remaining grasps. Redmon et al. [40] proposed a different network structure that directly regresses the grasping pose in a single step, making it faster and more accurate. Beyond RGB-D images, researchers have also explored point cloud-based approaches for grasp detection, which offer a different perspective on the 3D environment. Qi et al. [41] first proposed PointNet, which learns features directly from raw point cloud input, and Qin et al. [42] extended its use to predict grasping poses in 3D space. However, despite the promising results, these methods still face significant computational challenges, limiting their widespread adoption in industrial settings.

Moreover, since the introduction of AlexNet [43,44], 2D image object detection has become increasingly mature, with models such as Fast-RCNN [45,46] and YOLO [18,47]. Liu et al. [48] proposed a grasp-pose-detection method based on a cascaded convolutional neural network, using Mask R-CNN to extract object grasp features and candidate bounding boxes. Karaoguz et al. [49] employed transfer learning based on CNN object-detection architectures to achieve grasp pose detection from RGB images. However, deep learning methods based on 2D images primarily focus on recognizing everyday objects, while industrial environments are more complex.

Concealed object detection (COD) [20] aims to detect objects that are visually highly integrated with their surrounding environment. This technology has been widely applied in areas such as species conservation [50], medical image segmentation [51], and industrial defect detection [52]. In 2020, Fan et al. released the first large-scale public dataset, COD10K [20], advancing the field of concealed object detection. This release has inspired further research in related disciplines. For example, Mei [21] proposed a distraction-aware framework for camouflaged object segmentation, which has potential applications in identifying transparent materials in natural scenes as well. Similar issues exist in the operational scenarios of industrial robotic arms, where complex environments require target detection algorithms to identify objects with highly similar foreground and background. Currently, the main camouflaged object detection datasets are CAMO [25], CHAMELEON [53], and COD10K [20], primarily consisting of images from everyday scenes. Notably, however, the industrial sector still lacks relevant large-scale datasets. To bridge this gap, we contribute a novel dataset specifically tailored for concealed object detection in industrial settings, along with the development of effective object-detection algorithms tailored for industrial applications.

### 2.2. Bounding Box Regression

Bounding box regression (BBR) is a fundamental component of object-detection systems, which predicts the coordinates of bounding boxes around objects in an image. The effectiveness of BBR directly impacts the overall performance of object-detection models. Various loss functions have been proposed to improve the accuracy and robustness of BBR, including those introduced by Felzenszwalb [54] et al. (2009), Girshick [55] et al. (2014), Beery [56] et al. (2020), and Wu [57] et al. (2020). IoU (Intersection over Union) is the most widely used loss function, advantaged by its ability to accurately describe the match between the predicted box and the ground truth box. However, its limitation becomes evident when the overlap is zero, as it fails to accurately describe their positional relationship, especially the rotational relationship. To address this, Rezatofighi et al. proposed GIoU [28], which considers the containment relationship and spatial distribution between bounding boxes. However, GIoU loses effectiveness when the ground truth box completely covers the predicted box. To tackle this issue, Zheng et al. proposed DIoU [29], which improves scale inconsistency by considering the distance between the center points of bounding boxes. However, when the center point of the predicted box coincides with that of the ground truth box, DIoU degenerates into the original IoU. Further, Zheng et al. proposed CIoU [58], which simultaneously considers the center point distance and aspect ratio. However, the aspect ratio defined in CIoU is relative rather than absolute.

Xue Yang [31] proposed a method that utilizes a two-dimensional Gaussian distribution to model the problem of rotated bounding boxes. By calculating the Gaussian Wasserstein distance, this approach approximates the loss caused by the non-differentiable rotating IoU, aligning model learning with accuracy measurement and addressing the inconsistency in the loss. However, if the object to be detected is a square, the calculated Gaussian distribution will always be a normal distribution with a covariance of 0 regardless of the rotation, leading to a computed loss that is always 0 while the IoU varies.

Recognizing the limitations of previous Gaussian-based methods, particularly in handling square objects, we introduce G/2-ProbIoU, a novel approach that evaluates the overlap between two Gaussian-distributed bounding boxes. This method incorporates a carefully designed stretching operation specifically tailored to handle squares effectively. We further propose Cube-mAP, a new evaluation metric based on G/2-ProbIoU, to comprehensively assess the detection and recognition performance of rotated bounding boxes.

### 2.3. Attention Mechanisms

Attention mechanisms [59] represent a simple yet powerful approach to enhancing neural representations across a wide range of tasks. Channel attention mechanisms, such as SE blocks [60], leverage global average information to reweigh feature channels. Spatial attention modules, including GENet [61], GCNet [62], and SGE [63], bolster the network’s capacity to model contextual information through spatial masks. CBAM [64] and BAM [65] integrate both channel and spatial attention. Additionally, kernel selection emerges as an adaptive and efficient dynamic context modeling technique. CondConv [66] and dynamic convolution [67] utilize parallel kernels to adaptively aggregate features across multiple convolutional kernels.

SKNet [68] introduces multiple branches with different convolutional kernels and selectively combines them along the channel dimension. SCNet [69] innovates with self-calibrated convolutions, enhancing convolutional transformations at each layer. ResNet [70] proposes a modular architecture that applies channel attention to different network branches, leveraging their success in capturing cross-feature interactions and learning diverse representations.

Building upon the above works, LSKNet [37] presented a lightweight detection backbone network. By weighting features processed through large-kernel convolutions and spatially merging them, it dynamically adjusts spatial receptive fields, enabling better modeling of contextual information across various object ranges in remote sensing scenes. RepLKNet [71], as a purely CNN architecture, achieves a kernel size of 31 × 31 for large-kernel convolutions, significantly exceeding the commonly used 3 × 3. Research has shown that CNNs employing large-kernel convolutions possess more effective receptive fields compared to those with smaller kernels, subsequently introducing greater shape biases into the network. Furthermore, the integration of large-kernel convolutions with residual structures effectively boosts the model’s performance. The FPN [72] model leverages the intrinsic multi-scale, pyramid hierarchical structure of deep convolutional networks to construct feature pyramids with almost no additional cost. Drawing inspiration from these methods, we propose the CS-SKNet backbone network, which incorporates the benefits of large-kernel convolutions within a multi-scale pyramid-like structure. This design aims to expand the network’s receptive field and effectively capture strong-texture features within the scene.

## 3. WoodenCube Dataset

**Figure 2:**
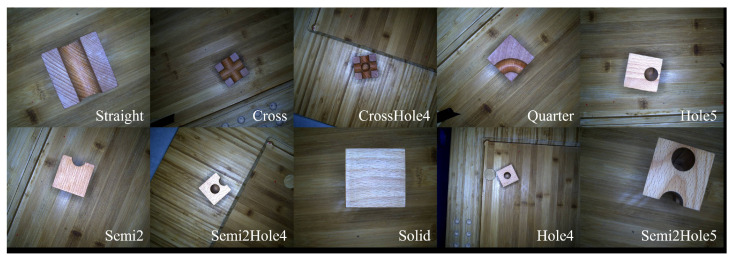
Single cube samples from WoodenCube. The wooden cube material is the same as the background; both are made of oak wood.

Figure 2 shows that the WoodenCube dataset in this paper provides data with varying heights, apertures, and exposures under various complex textures. Each cube is placed on a baseboard with a texture similar to the cube itself, and there is a significant resemblance between certain types, such as Semi2Hole4 and Semi2Hole5, which undoubtedly increases the difficulty of object detection. The dataset includes multiple views of each block, ensuring completeness and facilitating future work in block coding. This section will describe the main characteristics of the WoodenCube dataset and how we were able to construct such a dataset in a short period.

### 3.1. Data Collection

**Figure 3:**
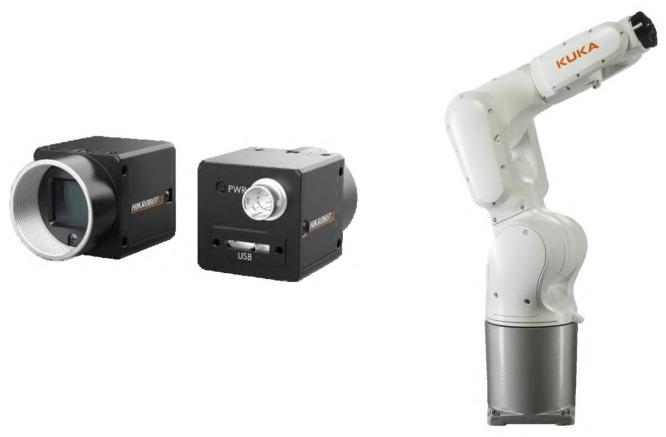
Data collection equipment. The left shows the MV-CS050-10UC industrial camera from Hikvision, while the right depicts the KUKA KR6 R900-2 robot.

To construct a high-quality dataset, we integrated a high-resolution 5-megapixel RGB camera with a precise six-axis robotic arm, as shown in Figure 3. The detailed specifications of the equipment are listed in Table 1. To ensure the comprehensive coverage and completeness of the dataset, we flexibly adjusted key parameters, including shooting distance, exposure, and gain through programming.

Furthermore, we incorporated various cubes as subjects, each having similar basic trajectory planes but possessing unique characteristics. Additionally, for every adjustment in shooting distance, we meticulously operated the camera’s focus adjustment ring to ensure each image captured met the highest clarity standards.

### 3.2. Data Annotation

**Figure 4:**
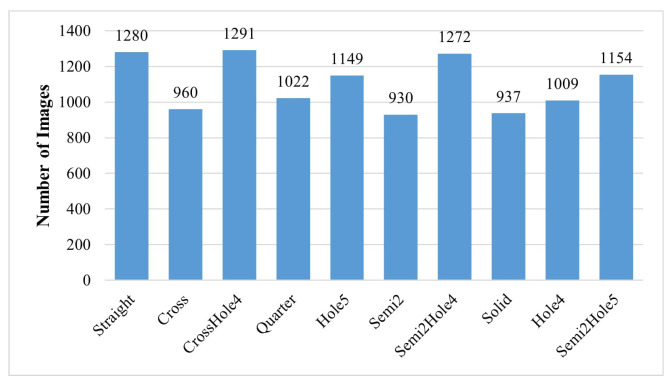
Class distribution of WoodenCube dataset.

Ultimately, we obtained the WoodenCube dataset, which comprises 5113 RGB images with a resolution of 2448 × 2048 pixels, covering 30 different target wooden cubes. Figure 4 illustrates the specific number of frames for each class and their distribution.

The annotation format of the WoodenCube dataset is “$x_{1}$, $y_{1}$, $x_{2}$, $y_{2}$, $x_{3}$, $y_{3}$, $x_{4}$, $y_{4}$, class, difficulty”. Currently, many publicly available datasets are annotated manually, a process that requires considerable manpower and resources. Moreover, due to the differences among annotators and their subjectivity, manual annotation introduces a significant amount of uncertainty. Therefore, we propose the use of the Segment Anything Model (SAM) segmentation algorithm for the semi-automatic annotation of the dataset. In the SAM segmentation algorithm [32], directly using the entire image as the segmentation scope does not yield the expected results and can result in significant errors.

**Figure 5:**
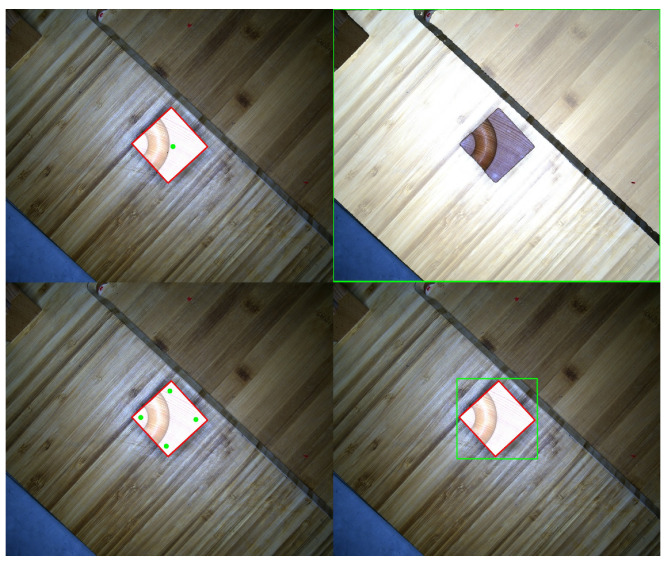
Compares the fitting effects of three auxiliary annotation methods. The two left images show annotation with 1 and 4 reference points, respectively; the top right image depicts the entire image as the reference area, and the bottom right image shows a green horizontal box as the reference area. The green annotated points and box are obtained through manual annotation, while SAM obtains the red box combined with computing the minimum bounding rectangle of the convex hull.

To ensure that the algorithm can achieve high-accuracy masks for specific objects, we first annotate several reference points on the image of wooden cubes. Although this improves the segmentation results, in scenes with strong textures, the edges of the wooden cubes and the background textures are very similar, leading to some errors in the segmented mask at the edges. Additionally, when only one reference point is annotated, it fails to guarantee complete coverage of the entire cube by the mask; while annotating multiple reference points yields better results, it significantly increases the annotation workload. Therefore, this paper proposes providing a horizontal bounding box to annotate the object in the image. Experimental results demonstrate that annotating a horizontal bounding box as the detection range for the object, prior to applying the SAM algorithm for segmentation, achieves the highest mask accuracy. Figure 5 illustrates the effects of the three methods above of assisted annotation using the SAM algorithm.

After obtaining a mask for a given wooden cube using the SAM algorithm, it is straightforward to comprehend that the essence of this mask is a collection of points. We first propose calculating the convex hull of this point set and then determining the minimum bounding rectangle of the convex hull to fit the rotation anchor box corresponding to the wooden cube.

**Figure 6:**
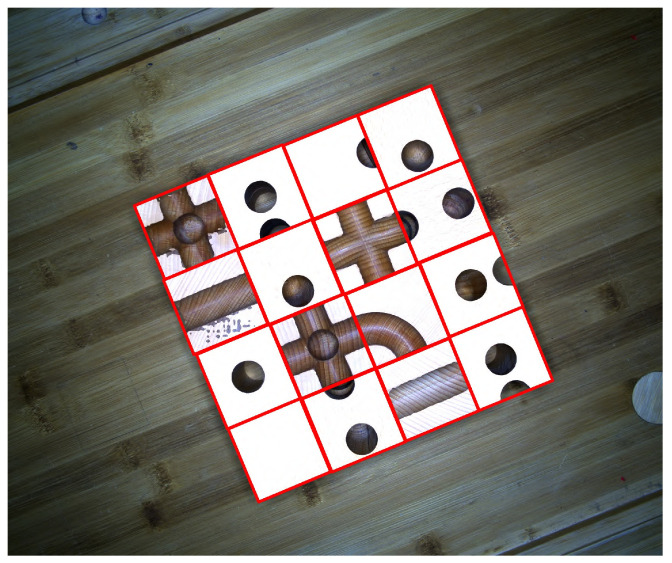
The influence of interfering texture points on the fitting of the resulting rotated anchor boxes.

The above-described method is a semi-automatic annotation approach based on segment-anything, specifically designed and employed in this paper. Although this approach significantly reduces the manpower and resources required for annotating the dataset, it is not without limitations. For instance, interference from the sides of the wooden cubes can markedly increase the error of the computed rotation anchor box. Furthermore, certain strong textures or interfering textures on the background or other surfaces of the wooden cubes can cause considerable displacement of the rotation anchor box, as illustrated in Figure 6. Despite employing a range of sophisticated post-processing techniques, such as denoising, it remains challenging to fully guarantee the accuracy of the annotations. Therefore, a swift manual screening is necessary to ensure the acquisition of high-quality dataset labels. Thus, in this industrial setting, a high-precision method for rotation object detection is urgently needed.

### 3.3. Cube-mAP Evaluation Method

**Figure 7:**
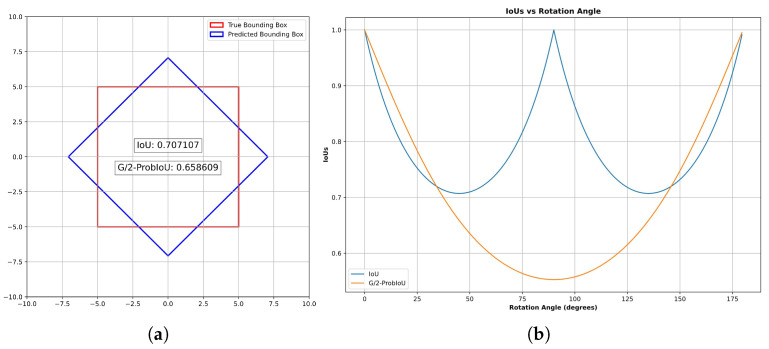
The performance of IoU and G/2-ProbIoU on class-square datasets containing mostly square-shaped objects. (**a**) The relationship between IoU and G/2-ProbIoU when two bounding boxes are rotated 45°with their centers overlapped. (**b**) The variation in IoU and G/2-ProbIoU with the rotation angle when the centers of the bounding boxes overlap.

Currently, mAP (mean average precision) is an important and commonly used evaluation metric in object detection. It first computes the overlap between predicted and ground truth bounding boxes using Intersection over Union (IoU [27]). A higher overlap indicates closer proximity to the ground truth box. Then, based on a given IoU threshold, it determines whether the bounding box is TP (true positive), FN (false negative), FP (false positive), or TN (true negative). The formula for IoU calculation is as follows:

(1) $$IoU=\frac{A\cap \tilde{A}}{A\cup \tilde{A}}$$

where *A* and $\tilde{A}$ represent the areas of the ground truth and predicted bounding boxes, respectively. In rotated object detection, as shown in Figure 7a, the distribution of the ground truth bounding box and the predicted bounding box is such that, when the centers of the two boxes coincide and their rotation angles differ by 45°, the IoU is already 0.7071. Generally, when the threshold is set at 0.7, the situation depicted in Figure 7b would be considered a true positive (TP). However, such a large rotation angle does not align with our expectations. Therefore, using mAP as the evaluation metric for rotated object detection is not sufficiently objective, especially for datasets containing mostly square-shaped rotated objects.

Inspired by Yang [31] and Jeffri M. Llerena [33], we model rotated bounding boxes as Gaussian distributions and utilize the G/2-ProbIoU metric to evaluate the overlap between two bounding boxes. An overview of the algorithm is presented in Algorithm 1. Firstly, we transform a rotated bounding box B(x,y,w,h,θ) into a two-dimensional Gaussian distribution N(m,Σ) [31,33]. Thus, evaluating the overlap between two rotated bounding boxes $B(x_{1},y_{1},w_{1},h_{1},\theta_{1})$, $B(x_{2},y_{2},w_{2},h_{2},\theta_{2})$, is equivalent to assessing the difference between two two-dimensional Gaussian distributions $N(m_{1},\Sigma_{1})$, $N(m_{2},\Sigma_{2})$. For the convenience of calculation, we can decompose the two-dimensional Gaussian distribution into an expression in terms of the principal axes through eigenvector decomposition. However, for the aforementioned WoodenCube dataset, its rotated bounding boxes exhibit strong square-like features, leading to Gaussian distributions close to isotropic, showing approximately circular shapes. In this case, the values of various IoUs tend to be relatively high, exceeding typical IoU thresholds. According to the original calculation method of mAP, they would be categorized as true positives (TP), neglecting other important factors such as rotation angles. Therefore, it may be purposeful to stretch the Gaussian distribution to prevent it from approaching an isotropic Gaussian distribution; this stretching operation is reversible. For simplicity, let us stretch the Gaussian distribution along the principal axis y direction by a factor of 1, resulting in the covariance matrix of the transformed Gaussian distribution. The formula below demonstrates how we diagonally orthogonalize the transformed covariance matrix.

(2) $$\begin{array}{cc} \Sigma \; & =\left[\begin{array}{cc} a & c\\ c & d \end{array}\right]=R_{\alpha }\left[\begin{array}{cc} \lambda_{1} & 0\\ 0 & 2\lambda_{2} \end{array}\right]R_{\alpha }^{T}\\ \; & =\left[\begin{array}{cc} \lambda_{1}\cos^{2}\alpha +2\lambda_{2}\sin^{2}\alpha & \frac{1}{2}\left(\lambda_{1}-2\lambda_{2}\right)\sin2\alpha \\ \frac{1}{2}\left(\lambda_{1}-2\lambda_{2}\right)\sin2\alpha & \lambda_{1}\sin^{2}\alpha +2\lambda_{2}\cos^{2}\alpha \end{array}\right] \end{array}$$

where $R_{\alpha }$ = $\left[\begin{array}{cc} \cos\alpha & -\sin\alpha \\ \sin\alpha & \cos\alpha \end{array}\right]$ represents the two-dimensional rotation matrix, $\lambda_{1},2\lambda_{2}$ represent the eigenvalues of the covariance matrix Σ, and they are also the representations of *a* and *b* in the new coordinates. Since we have already transformed the original two-dimensional Gaussian distribution into an expression along the principal axes, the method for computing the covariance matrix of the rotated bounding box is consistent with that of the horizontal bounding box. Here, we will derive the method using the rotated bounding box as an example. For the rotated bounding box, the region Ω degenerates into a rectangular region with a center point at $\left(x_{c},y_{c}\right)$ and widths and heights, respectively, denoted as *w* and 2h. We integrate along the principal axes to compute the covariance matrix for *x* and *y*.

(3) $$\begin{array}{c} \Sigma =\frac{1}{wh}\int_{-h}^{h}\int_{-\frac{w}{2}}^{\frac{w}{2}}\left[\begin{array}{cc} x^{2} & xy\\ xy & y^{2} \end{array}\right]dxdy=\left[\begin{array}{cc} \frac{w^{2}}{6} & 0\\ 0 & \frac{2h^{2}}{3} \end{array}\right] \end{array}$$

Thus, we have

(4) $$a=\frac{w^{2}}{6},b=\frac{2h^{2}}{3},c=0$$

**Algorithm 1.** G/2-ProbIoU

**Algorithm Overview:**
A Method for Calculating IoU with Stretched Gaussian Distribution.**Input:**$B(x_{i},y_{i},w_{i},h_{i},\theta_{i}),i=1,2$.**Output:** G/2ProbIoU.
 1:$a_{i}^{\prime }=\frac{w_{i}^{2}}{6},b_{i}^{\prime }=\frac{2h_{i}^{2}}{3},c_{i}^{\prime }=0$. 2:$\Sigma =\left[\begin{array}{cc} a_{i} & c_{i}\\ c_{i} & b_{i} \end{array}\right]=R_{\theta }\left[\begin{array}{cc} a_{i}^{\prime } & c_{i}^{\prime }\\ c_{i}^{\prime } & b_{i}^{\prime } \end{array}\right]R_{\theta }^{T}$. 3:$\Sigma =\left[\begin{array}{cc} a_{i}^{\prime }\cos^{2}\theta_{i}+b_{i}^{\prime }\sin^{2}\theta_{i} & \left(a_{i}^{\prime }-b_{i}^{\prime }\right)\sin\theta_{i}cos\theta_{i}\\ \left(a_{i}^{\prime }-b_{i}^{\prime }\right)\sin\theta_{i}cos\theta_{i} & a_{i}^{\prime }\sin^{2}\theta_{i}+b_{i}^{\prime }\cos^{2}\theta_{i} \end{array}\right]$. 4:$B_{1}=\frac{1}{4}\cdot \frac{\left(a_{1}+a_{2}\right){\left(y_{1}-y_{2}\right)}^{2}+\left(b_{1}+b_{2}\right){\left(x_{1}-x_{2}\right)}^{2}}{\left(a_{1}+a_{2}\right)\left(b_{1}+b_{2}\right)-{\left(c_{1}+c_{2}\right)}^{2}}$. 5:$B_{2}=\frac{1}{2}\frac{\left(c_{1}+c_{2}\right)\left(x_{2}-x_{1}\right)\left(y_{1}-y_{2}\right)}{\left(a_{1}+a_{2}\right)\left(b_{1}+b_{2}\right)-{\left(c_{1}+c_{2}\right)}^{2}}$. 6:$B_{3}=\frac{1}{2}\cdot \ln\left(\frac{\left(a_{1}+a_{2}\right)\left(b_{1}+b_{2}\right)-{\left(c_{1}+c_{2}\right)}^{2}}{4\sqrt{\left(a_{1}b_{1}-c_{1}^{2}\right)\left(a_{2}b_{2}-c_{2}^{2}\right)}}\right)$. 7:$D_{B}=B_{1}+B_{2}+B_{3}$. 8:$BC=e^{-D_{B}}$. 9:$H\left(p,q\right)=\sqrt{1-BC(p,q)}$.10:G/2ProbIoU(p,q)=1−H(p,q).


Two rotated bounding boxes are transformed into two Gaussian distributions, denoted as $p∼N(\mu_{1},\Sigma_{1})$ and $q∼N(\mu_{2},\Sigma_{2})$. According to the above theory, we stretch the Gaussian distributions *p* and *q*, keeping the mean vectors $\mu_{1}$ and $\mu_{2}$ unchanged.

Jeffri M. Llerena [33] uses the Bhattacharyya coefficient to measure the overlap between two distributions, thus deriving an analytical expression for the Bhattacharyya coefficient concerning the Bhattacharyya distance in the two-dimensional case. Subsequently, the Hellinger distance is employed to gauge the similarity between two probability distributions. Under the theoretical framework of this paper, we can similarly utilize the Hellinger distance to measure the similarity between Gaussian distributions *p* and *q* and obtain analogous analytical expressions. The Bhattacharyya coefficient BC can be used to quantify the overlap between distributions *p* and *q*, and the Bhattacharyya distance $D_{B}$ between distributions *p* and *q* can be derived from the logarithmic definition of the Bhattacharyya coefficient.

(5) $$\begin{array}{c} \left\{\begin{array}{c} BC\left(p,q\right)=\int_{R}^{2}\sqrt{p(x)q(x)}dx\\ D_{B}\left(p,q\right)=-\ln BC(p,q) \end{array}\right. \end{array}$$

Then, an analytical expression for the Bhattacharyya distance $D_{B}$ can be obtained.

(6) $$D_{B}\;=\;\frac{1}{8}\cdot {\left(\mu_{1}-\mu_{2}\right)}^{T}\Sigma^{-1}\left(\mu_{1}-\mu_{2}\right)\;+\;\frac{1}{2}\cdot \ln\left(\frac{\left|\Sigma \right|}{\sqrt{\left|\Sigma_{1}\right|\left|\Sigma_{2}\right|}}\right)$$

where $\Sigma =\frac{1}{2}\cdot \left(\Sigma_{1}+\Sigma_{2}\right)$. Thus, derive an analytical expression for the Bhattacharyya coefficient in terms of the Bhattacharyya distance.

(7) $$BC=e^{-D_{B}}$$

We use the Hellinger distance to measure the similarity between Gaussian distributions *p* and *q*, which can be regarded as the complement of the square root of the Bhattacharyya coefficient, and its values range from 0 to 1.

(8) $$H\left(p,q\right)=\sqrt{1-BC(p,q)},\;H\left(p,q\right)>0$$

Then, we use 1−H(p,q) to measure the similarity between Gaussian distributions p(x) and q(x), denoted as G/2ProbIoU.

(9) G/2ProbIoU(p,q)=1−H(p,q)

According to Figure 7b, for two squares with overlapping centers, the IoU curve is mostly above the G/2-ProbIoU curve, with the lowest point of the IoU curve being 0.1 higher than G/2-ProbIoU. The period of IoU variation concerning rotation angle is π/2, while for G/2-ProbIoU, it is π, which also reflects the idea of stretching Gaussian distributions for G/2-ProbIoU.

Returning to the distribution of true bounding boxes and predicted bounding boxes as shown in Figure 7a, when the centers of the two bounding boxes coincide, and the rotation angles differ by 45°, the IoU is 0.7071, while G/2-ProbIoU is only 0.6586. At a typical threshold of 0.7, the scenario depicted in Figure 7a would not be counted as a true positive (TP), which aligns with the expected behavior for detecting rotated objects resembling squares.

Below, based on mAP and the previously introduced G/2-ProbIoU, we construct a new evaluation metric called Cube-mAP. The calculation method is as follows:

First, compute the G/2-ProbIoU value between the predicted bounding box and the ground truth bounding box. Next, determine whether the predicted bounding box is categorized as TP (True Positive), FN (False Negative), FP (False Positive), or TN (True Negative) based on a given G/2-ProbIoU threshold. In this case, we set the threshold to 0.7, meaning that if the G/2-ProbIoU of the predicted box is greater than 0.7, it is considered a TP; otherwise, it is an FP. FP represents the number of falsely detected negative samples, and FN represents the number of missed positive samples. Then, we calculate the P (Precision) and R (Recall) values for each category using the following formulas:

$$\mathrm{Precision}=\frac{\mathrm{TP}}{\mathrm{TP}+\mathrm{FP}},\;\mathrm{Recall}=\frac{\mathrm{TP}}{\mathrm{TP}+\mathrm{FN}}$$

Subsequently, we plot the corresponding P-R curve for each category. Using the 11-point interpolation method, we calculate the AP for each category. This involves taking the maximum Precision value at 11 points of Recall from 0 to 1 (0.0, 0.1, 0.2, …, 1.0) and averaging these values:

$$\mathrm{AP}=\frac{1}{11}\sum_{r\in \{0.0,0.1,...,1.0\}}\max_{\tilde{r}\geq r}\mathrm{Precision}\left(\tilde{r}\right)$$

Finally, the Cube-mAP is obtained by averaging the AP values of all categories.

## 4. CS-SKNet Architecture

**Figure 8:**
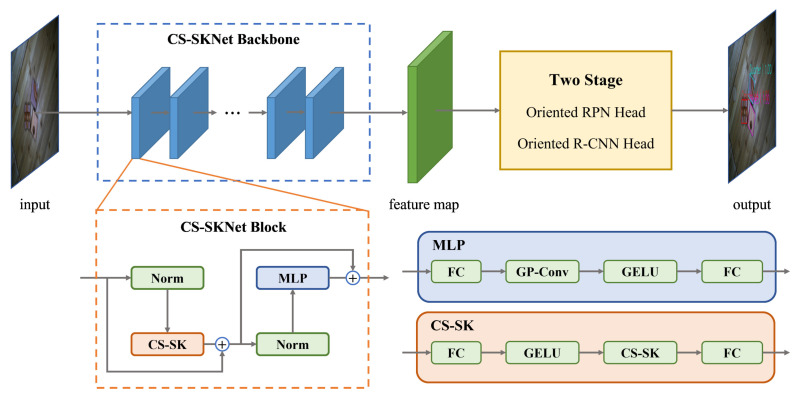
Overall framework of CS-SKNet.

The overall architecture of CS-SKNet is shown in Figure 8. It is composed of a series of repeated CS-SKNet blocks, where each CS-SKNet block consists of two key sub-blocks: the CS selection sub-block and the multi-layer perceptron (MLP) sub-block, drawing inspiration from LSKNet [37], RepLKNet [71], and FPN [72].

**Figure 9:**
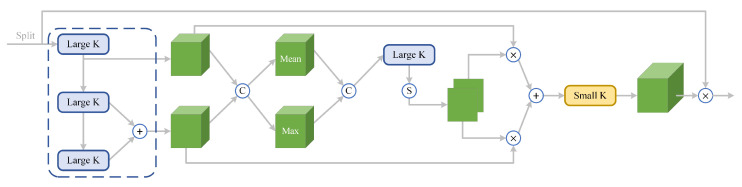
CS selection sub-block.

As shown in Figure 9, the role of the CS selection sub-block is to dynamically adjust the network’s receptive field as needed, which is achieved through the CS-SK core module. This core module comprises a series of pyramid-like large-kernel convolutions and spatial kernel selection mechanisms, which will be detailed later. The MLP sub-block, on the other hand, is used for channel fusion and feature refinement. It consists of operations, including the first fully connected layer, group convolution layer, GELU activation function, and the second fully connected layer. Combining these operations effectively enhances the quality and diversity of feature representation.

### 4.1. Multi-Scale Pyramid-like Large-Kernel Convolution Block

In order to achieve high-precision localization and classification of goods in industrial scenes using the WoodenCube dataset proposed in this paper, we aim to fully utilize the extensive contextual information in the images to be detected. Therefore, we propose a multi-scale pyramid-like large-kernel convolution in the CS selection sub-block. Unlike the FPN [72] pyramid hierarchical structure, this pyramid-like large-kernel convolution does not change the feature maps’ size or the number of channels at each layer. Although the dimensions of the feature maps remain unchanged, under the influence of this multi-scale large-kernel convolution block, we can generate large receptive fields at different scales, which facilitates subsequent spatial kernel selection.

For convolutional layers, we know that the theoretical receptive field size calculation formula is as follows

(10) $$\begin{array}{c} \left\{\begin{array}{c} RF_{0}=1\\ RF_{i}=RF_{i-1}+\left(k_{i}^{\prime }-1\right)S_{i},\;i>=1 \end{array}\right. \end{array}$$

where $k_{i}^{\prime }$ represents the effective kernel size and $S_{i}$ denotes the product of the strides from the 1st to the *i*th layer.

(11) $$\begin{array}{cc} k_{i}^{\prime } & =k_{i}+\left(k_{i}-1\right)\left(d_{i}-1\right) \end{array}$$

(12) $$\begin{array}{cc} S_{i} & =\prod_{j=1}^{i}s_{j}=S_{i-1}\cdot s_{i} \end{array}$$

where $k_{i}$ represents the actual size of the convolution kernel, $d_{i}$ represents the dilation rate of the *i*-th convolutional layer, and $s_{i}$ represents the stride of the *i*-th convolutional layer.

In the CS selection sub-module, we employ a pyramid-shaped large-kernel convolution, followed by the addition of the two feature maps produced by the final large-kernel convolution before outputting them to the subsequent model. This implies that the theoretical receptive field is not equal to the latter after the final layer of large-kernel convolution. This design has two advantages. Firstly, simplifying the original *N* feature maps into two feature maps effectively reduces the complexity of the model, decreases the number of parameters, and enhances the model’s computational speed. Secondly, it enables the full integration of information across different receptive fields, thereby obtaining features with richer contextual information.

### 4.2. A Plug-and-Play MLP Based on Group Convolution

**Figure 10:**
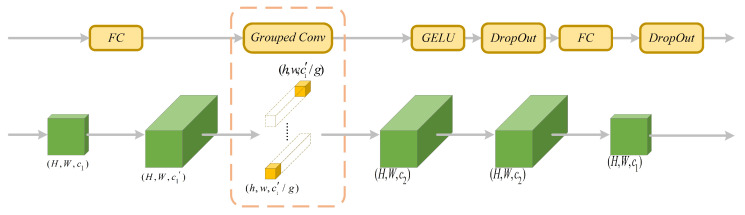
The structure of multi-layer perceptron.

The MLP sub-module constructed in this paper comprises a first fully connected layer, a group convolutional layer, a GELU activation function, and a second fully connected layer. Figure 10 illustrates the structure of the MLP sub-module. In the MLP sub-module, we incorporate fully connected layers before and after the sub-module to better capture abstract features in the input data and enhance the model’s expressive capability. Introducing fully connected layers before and after the feature map can reduce the computational cost of this sub-module, improve the model’s generalization ability, and ensure that the scale and number of channels of the feature map remain unchanged before and after entering the sub-module, thus achieving plug-and-play functionality.

The core of MLP based on group convolution lies in integrating group convolution into the MLP sub-module. Group convolution was first introduced in AlexNet due to hardware resource constraints at the time of training. Training the entire AlexNet network on a single GPU was not feasible. Therefore, the authors distributed the convolution operation across multiple GPUs and merged the results from these GPUs, thus giving rise to group convolution. In the MLP sub-module of this paper, the feature maps with dimensions *H*, *W*, and $c_{1}^{\prime }$ after the first fully connected layer are input into the group convolution layer.

We maintain the dimensions *H*, *W*, and $c_{1}^{\prime }$ of the input feature maps while dividing them into *g* groups based on the number of channels, resulting in each group having dimensions *H*, *W*, and $c_{1}^{\prime }/g$. Correspondingly, the convolutional kernels remain the same size, with input channels of $c_{1}^{\prime }/g$, and each group has $c_{2}/g$ convolutional kernels. We apply independent convolutional kernels to each group of feature maps, perform standard convolution operations, and concatenate the resulting g groups of feature maps with dimensions *H*, *W*, and $c_{2}/g$ to obtain an output feature map with dimensions *H*, *W*, and $c_{2}$. Through the use of group convolution, the number of model parameters in the MLP sub-module can be reduced by a factor of *g*.

(13) $$H\cdot W\cdot \frac{c_{1}^{\prime }}{g}\cdot \frac{c_{2}}{g}\cdot g=\frac{HWc_{1}^{\prime }c_{2}}{g}$$

## 5. Experiments

### 5.1. Datasets and Implementation Details

After collecting and annotating the WoodenCube dataset, we divided the dataset into training, validation, and test sets using an 8:1:1 ratio. All experiments were conducted on an RTX 4090 GPU with a batch size of 4. During the training phase, all images in WoodenCube were resized to 1024 × 1024 pixels. The evaluation was performed on the test set of WoodenCube, and all reported FLOPs in this paper were calculated based on input images of size 1024 × 1024.

To validate the generality of our approach, we also conducted experiments on the DOTAv1.0 [74] public dataset. The DOTAv1.0 dataset consists of 2806 aerial images captured by various sensors and platforms, with image sizes ranging from 800 to 4000 pixels. The images display objects of various scales, orientations, and shapes, annotated by domain experts, covering 15 common object categories. The images in the DOTAv1.0 dataset are fully annotated, totaling 188,282 instances, with each instance marked using quadrilaterals. The training, validation, and test sets of the DOTAv1.0 dataset consist of 1411, 458, and 937 images, respectively. All images are cropped to a size of 1024 × 1024 pixels. For multi-scale training, images are resized to 0.5×, 1.0×, and 1.5×of their original size before cropping, with a 500-pixel overlap.

The evaluation model in this study is built based on the Oriented RCNN [75] framework, implemented under the mmrotate [76] framework, and all models are trained on the training and validation sets and tested on the test set. Unlike LSKNet [37], this experiment did not adopt the pre-training backbone network strategy but instead trained these models from scratch, with an initial learning rate set to 0.0002 and weight decay to 0.05. During training, we utilized horizontal, vertical, and diagonal flips, as well as random polygon rotation as data augmentation methods, and employed exponential moving average (EMA) to weight-smooth the data and model weights. This paper evaluates the WoodenCube dataset using the Cube-mAP evaluation metric.

### 5.2. Main Result

**Figure 11:**
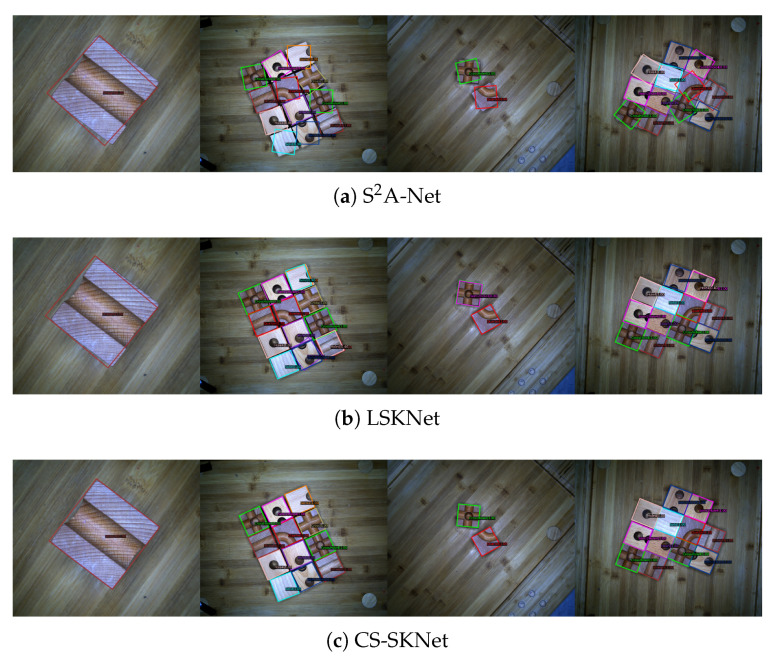
Visualization comparison of three methods on the WoodenCube dataset. (**a**–**c**) Results corresponding to the S2A-Net, LSKNet, and CS-SKNet models.

In this section, we present the experiments conducted on the WoodenCube dataset and the DOTAv1.0 dataset to demonstrate the Cube-mAP metric’s rationality and the CS-SKNet model’s feasibility. We compared our approach with several current state-of-the-art models and mainstream frameworks, all of which are well-established and widely recognized in the field. As shown in Table 2, Cube-mAP provides a highly accurate evaluation on the WoodenCube dataset, whereas mAP often yields a score of 100 in many frameworks, which is not listed in the table. From the visualization results in Figure 11, it can be observed that the CS-SKNet model can accurately detect the rotational components of the wooden cubes, whereas the S^2^A-Net [77] and LSKNet [37] models, although proficient in locating the center points of the cubes, still have room for improvement in detecting rotational components.

**Figure 12:**
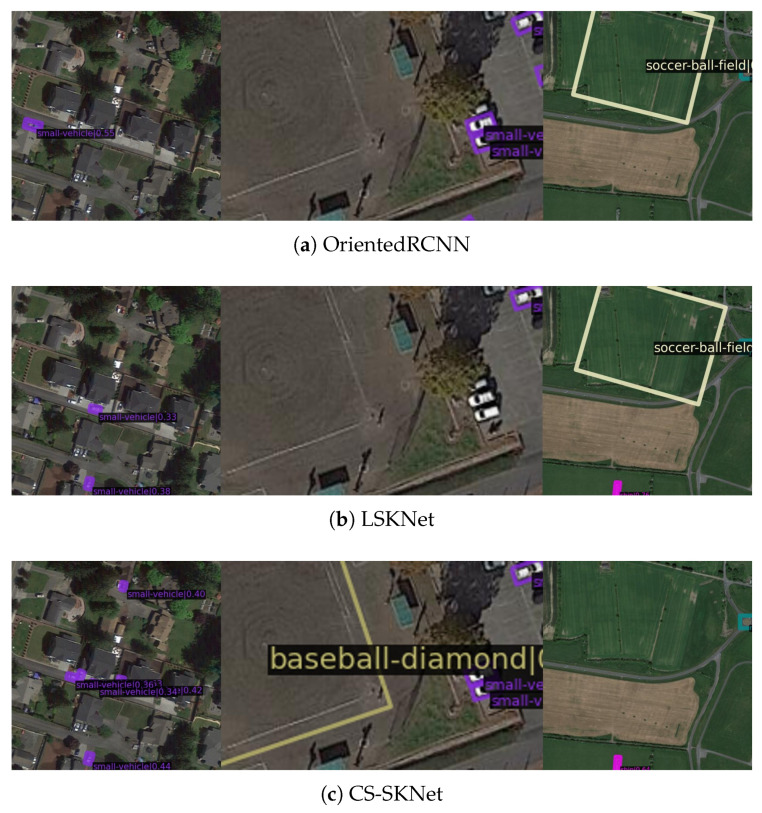
Visualization comparison of three methods on the DOTAv1.0 dataset. (**a**–**c**) Results corresponding to the OrientedRCNN, LSKNet, and CS-SKNet models.

Using the DOTAv1.0 dataset, we compared our approach with 12 state-of-the-art methods, as shown in Table 3. Our CS-SKNet achieved an mAP of 79.17%. It is worth noting that CS-SKNet achieved an inference speed of 31.9 FPS on a single RTX 4090 for images sized at 1024 × 1024. From the visualization result of Figure 12 on the DOTAv1.0, we can observe that the CS-SKNet model proposed in this paper performs well in detecting small and large objects. Furthermore, the CS-SKNet model, equipped with a large receptive field, can effectively capture environmental information surrounding the targets, thereby minimizing false detections.

Overall, the CS-SKNet model and Cube-mAP metric provide an effective solution for object detection tasks in industrial scenarios and advance the field’s development. CS-SKNet maintains high accuracy while keeping parameter counts low and inference speeds high, making it highly promising for practical applications.

### 5.3. Ablation Study

#### 5.3.1. The Rationality of Cube-mAP

To evaluate the universality of the Cube-mAP metric, we conducted performance evaluations on the WoodenCube dataset using different detection frameworks. This includes the two-stage detection framework Oriented RCNN [75] and the one-stage detection frameworks S^2^A-Net [77] and R3Det [36], among other currently popular frameworks.

The results in Table 2 indicate that the Cube-mAP metric performs well in evaluating the detection performance on square datasets. Due to mAP yielding perfect scores of 100 in many frameworks, we did not list it in the table. It fails to accurately assess the detection performance of square-like objects, particularly in datasets with single backgrounds, such as our WoodenCube dataset. Thereby, the Cube-mAP metric provides a meaningful evaluation method for real-time robotic assembly of wooden cubes in scenarios like the one presented in this paper, where robots are tasked with picking up and assembling cubes in an environment where the foreground and the background are similar.

#### 5.3.2. Large Kernel Decomposition

From the results in Table 4, we explored several different strategies for decomposing large kernels to compare their impact on frames per second (FPS) and Cube-mAP. We found that the optimal solution for our proposed CS-SKNet model is to decompose the large kernel into three grouped convolution kernels and concatenate them in series.

This decomposition method maintains detection accuracy while also ensuring computational efficiency. it can be seen that the best solution for CS-SKNet on the WoodenCube dataset proposed in this paper is to decompose the large kernel into three grouped convolution kernels in series. Table 5 shows that too small or too large receptive fields will affect the performance of the CS-SKNet model, and in the WoodenCube dataset, a receptive field size of approximately 63 was determined to be the most effective, with a rate of 33.3 frames per second. This is sufficient to meet the needs of industrial robotic arms proposed in this paper for gripping scenarios.

#### 5.3.3. The Feasibility of CS-SKNet

To validate the performance improvements of the proposed CS-SKNet under different frameworks, we conducted a large number of experiments on the WoodenCube dataset and DOTAv1.0 dataset. The focus of the experiment was to compare the performance of CS-SKNet with the classic ResNet-50 [70] and ResNet-101 [70] backbone network, as shown in Table 2. In the WoodenCube dataset, we compared CS-SKNet with ResNet-50 in three detection frameworks: R3Det [36], Gliding Vertex [34], and Rotated FCOS [35]. For instance, in the Rotated FCOS framework, CS-SKNet achieves a 12% improvement in Cube-mAP compared to ResNet-50 and a 15% improvement over ResNet-101. Moreover, the increase in parameters and FLOPs across different detection frameworks is minimal compared to ResNet-101, demonstrating CS-SKNet’s adaptability and efficiency in multi-faceted wooden block scenarios.

In the DOTAv1.0 dataset, we also compared the detection performance of CS-SKNet and ResNet-50, as shown in Table 6. The results indicate that CS-SKNet significantly outperforms ResNet-50 on this dataset. Across all detection frameworks, CS-SKNet’s mAP is consistently about 4% higher than ResNet-50, with only a minimal increase in parameters and FLOPs.

## 6. Conclusions

We propose the WoodenCube dataset, comprising 5113 industrial scene images with similar foreground and background, featuring 10 different types of building blocks. Each image has been densely annotated with object-level categories, bounding boxes, and rotation angles. To facilitate the creation and annotation of this dataset, we introduced a semi-automatic annotation method, CS-SAM, which annotates a horizontal bounding box as the detection range for the object. Additionally, for near-square objects, we innovatively proposed the G/2-ProbIoU and Cube-mAP evaluation metrics, effectively addressing the issue of zero covariance in square Gaussian distributions. Furthermore, the multi-scale pyramid large-kernel convolution structure, CS-SKNet, designed in this study, expands the network’s receptive field to capture strong texture features in the scene, achieving high-precision localization and classification on the WoodenCube dataset. To further test the model’s generalization capability, we conducted extensive experiments on the DOTAv1.0 dataset, achieving a mAP of 79.17% while maintaining a low parameter count and computational load (FLOPs).

## Figures and Tables

**Table 1:** Equipment conditions.

Equipment	Details
Industrial camera	The MV-CS050-10UC, a second-generation industrial-line scan RGB camera, utilizes Sony’s IMX264 CMOS chip with a resolution of 2448 × 2048. It transmits uncompressed images in real-time via a USB 3.0 interface, with a maximum frame rate of up to 60 fps.
Robot	The KUKA KR6 R900 sixx six-axis robot weighs approximately 52 kg, with a maximum payload capacity of 6 kg. It has a maximum motion range of 901.5 mm and a pose repetition accuracy (ISO 9283 [73]) of ±0.03 mm.

**Table 2:** Comparison of CS-SKNet, ResNet-50 [70], and ResNet-101 [70] backbones under different detection frameworks on WoodenCube.

Frameworks	Backbone	Cube-mAP	Params (M)	FLOPs (G)
Oriented RCNN [75]	ResNet-50	**75.38**	**41.14**	**211.4**
	ResNet-101	72.89	60.13	289.32
	**CS-SKNet**	74.13	51.59	292.9
Rotated Faster RCNN [46]	ResNet-50	**72.90**	**41.13**	**211.3**
	ResNet-101	71.92	60.13	289.19
	**CS-SKNet**	70.44	51.59	294.6
R3Det [36]	ResNet-50	75.69	**41.90**	**335.7**
	ResNet-101	79.12	60.78	411.12
	**CS-SKNet**	**79.60**	48.61	419.5
Gliding Vertex [34]	ResNet-50	70.87	**41.14**	**211.3**
	ResNet-101	70.96	60.13	289.19
	**CS-SKNet**	**71.53**	51.59	294.6
Rotated FCOS [35]	ResNet-50	47.92	**31.91**	**206.7**
	ResNet-101	44.52	50.9	284.55
	**CS-SKNet**	**60.75**	42.41	293.2
S^2^A-Net [77]	ResNet-50	**59.24**	**38.60**	**197.6**
	ResNet-101	56.86	57.57	275.01
	**CS-SKNet**	57.26	45.31	281.4

Note: Bold indicates the best performance.

**Table 3:** Comparison with state-of-the-art methods on the DOTA-v1.0 dataset with multi-scale training and testing.

Method	mAP	# P(M)	FLOPs (G)	PL	BD	BR	GTF	SV	LV	SH	TC	BC	ST	SBF	RA	HA	SP	HC
R3Det [36]	76.47	41.9	336	89.8	83.77	48.11	66.77	78.76	83.27	87.84	90.82	85.38	85.51	65.57	62.68	67.53	78.56	72.62
CFA [78]	76.67	-	-	89.08	83.20	54.37	66.87	81.23	80.96	87.17	90.21	84.32	86.09	52.34	69.94	75.52	80.76	67.96
DAFNe [79]	76.95	-	-	89.4	**86.27**	53.70	60.51	**82.04**	81.17	**88.66**	90.37	83.81	**87.27**	53.93	69.38	75.61	**81.26**	70.86
S^2^A-Net * [77]	71.41	38.6	198	88.32	74.50	49.39	72.84	78.36	80.48	87.04	90.83	74.66	83.17	49.64	60.50	70.27	66.00	45.17
SCRDet [80]	72.61	-	-	89.98	80.65	52.09	68.36	68.36	60.32	72.41	90.85	**87.94**	86.86	65.02	66.68	66.25	68.24	65.21
RoT Trans * [81]	75.57	55.1	225	87.60	82.12	55.18	73.93	77.05	82.35	87.50	90.85	78.62	84.36	60.86	58.48	76.78	74.98	62.80
G.V. [34]	75.02	41.1	198	89.64	85.00	52.26	77.34	73.01	73.14	86.82	90.74	79.02	86.81	59.55	**70.91**	72.94	70.86	57.32
Oriented RCNN * [75]	75.05	41.1	211	88.49	79.14	53.32	77.34	76.93	82.67	87.98	90.85	77.41	83.35	59.56	64.26	75.48	68.83	60.14
CenterMap [82]	76.03	41.1	198	89.83	84.41	54.60	70.25	77.66	78.32	87.19	90.66	84.89	85.27	56.46	69.23	74.13	71.56	66.06
CSL [83]	76.17	37.4	236	**90.25**	85.53	54.64	75.31	70.44	73.51	77.62	90.84	86.15	86.69	**69.6**	68.04	73.83	71.10	68.93
LSKNet * [37]	78.96	**31.0**	**174**	89.03	83.12	56.76	79.32	78.40	84.66	87.97	**90.91**	85.62	85.04	63.68	65.89	77.94	79.31	**76.73**
**CS-SKNet (Ours)**	**79.17**	51.3	293	88.77	83.56	**56.87**	**80.71**	78.93	**84.68**	87.93	90.88	85.29	87.25	64.22	66.09	**77.96**	79.33	75.15

Note: * represents the results reproduced in this paper. Bold indicates the best performance.

**Table 4:** The effects of the number of decomposed large kernels on the inference FPS and mAP were examined through experiments conducted on the WoodenCube dataset.

(k,d) Sequence	Num.	RF	FPS	Cube-mAP
(47,1)	1	47	23.1	72.30
(7,1), (9,5)	2	47	**38.3**	72.26
(5,1), (7,3), (9,3)	3	47	33.7	**72.62**

Note: Bold indicates the best performance.

**Table 5:** The effectiveness of the key design components of CS-SKNet was examined through experiments conducted on the WoodenCube dataset.

($k_{1}$,$d_{1}$) Sequence	($k_{2}$,$d_{2}$) Sequence	($k_{3}$,$d_{3}$) Sequence	RF	FPS	Cube-mAP
(3,1)	(5,2)	(7,3)	29	**34.7**	71.58
(3,1)	(5,3)	(7,4)	39	**34.7**	72.65
(5,1)	(7,3)	(9,3)	47	33.7	72.62
(5,1)	(7,4)	(9,3)	53	32.9	73.26
(7,1)	(9,2)	(11,4)	63	33.3	**74.13**
(7,1)	(9,3)	(11,4)	71	33.0	72.80

Note: Bold indicates the best performance.

**Table 6:** Comparison of CS-SKNet and ResNet-50 [70] backbones under different detection frameworks on DOTAv1.0.

Frameworks	Backbone	mAP	Params (M)	FLOPs (G)
Oriented RCNN [75]	ResNet-50	75.05	**41.14**	**211.4**
	**CS-SKNet**	**79.17**	51.33	292.9
RoI Trans [81]	ResNet-50	75.57	**55.13**	**225.3**
	**CS-SKNet**	**78.74**	76.32	306.8
S^2^A-Net [77]	ResNet-50	71.41	**38.60**	**197.6**
	**CS-SKNet**	**76.84**	45.31	281.4
R3Det [36]	ResNet-50	69.55	**41.90**	**335.7**
	**CS-SKNet**	**75.08**	48.61	419.5

Note: Bold indicates the best performance.

## Data Availability

The data are contained within the article.
